# Cross-Cultural Adaptation of the Arabic Version of the Waterloo Footedness Questionnaire-Revised to Assess Footedness in Arabic-Speaking Adults

**DOI:** 10.7759/cureus.44421

**Published:** 2023-08-31

**Authors:** Mishal M Aldaihan

**Affiliations:** 1 Department of Rehabilitation Sciences, College of Applied Medical Sciences, King Saud University, Riyadh, SAU

**Keywords:** questionnaire translation and adaptation, translation process, translation, wfq-r, waterloo footedness questionnaire—revised, arabic, cross-cultural adaptation, footedness, waterloo, psychometric properties

## Abstract

Purpose: The goal of this research is to translate and analyze the psychometric properties of the Arabic version of the Waterloo Footedness Questionnaire-Revised (WFQ-R).

Materials and methods: Two native Arabic speakers created separate forward translations, which were then merged. Two different multilingual translators were used to translate it back into English from the synthetic version. Experts were gathered for a discussion on how to improve the localization and adaptation processes. A group of specialists was convened to analyze the localization and modification procedures. We now have the Arabic version of the WFQ-R which is "WFQ-R-Ar". Two hundred and ninety Arabic-speaking adults over the age of 18 were surveyed to evaluate the WHQ-R-Ar's characteristics (internal consistency, test-retest reliability, and construct validity).

Results: The WFQ-R-Ar had no collisions with the ground or the sky. The factor analysis showed that the construct validity of the WFQ-R-Ar was dependent on a single factor. The WFQ-R-Ar also has excellent internal consistency, with a Cronbach's alpha of 0.93. The reliability of the examinations was examined, and it was found to have an intraclass correlation value of 0.94.

Conclusion: The WFQ-R-Ar may be relied on to provide accurate results when used to evaluate footedness in Arabic-speaking society.

## Introduction

One of the behavioral predictors often used to determine cerebral lateralization is foot preference, also known as footedness [1]. Footedness, which in the context of the foot is comparable to handedness [2], is most usually associated with a preference for one foot over another when participating in foot-related sports. Footedness, handedness, eariness, and eyedness laterality preferences have been linked in studies on motor behavior and neuropsychology [3-5]. Earlier studies have shown a correlation between hand or foot preferences and various brain asymmetries, including linguistic organization, affective perception, and visuospatial abilities [6-8].

Research has looked into footedness and how it relates to postural control, gait, or motor abilities [9]. According to a review by Sadeghi and colleagues [10], differences in lower limb anatomy have been discovered to cause asymmetries in gait between the left and right legs, even in healthy, able-bodied people. In the study by Tate and colleagues [11], in young athletes, the vastus medialis muscle was greater on their dominant side. The vast lateralis muscle was greater in the non-dominant limb.

Evidence suggests that leg preference may influence postural control [12-14]. For instance, the preference among people with mild to severe hemiparesis after a stroke determines which lower limb is usually used for support during an upright stance. For those with more severe disabilities, the convenience field determines the supporting lower extremity [15, 16]. Given the findings from the research listed above, rather than single-item questions, lateral preference should be evaluated using multi-item inventories (e.g., writing for handedness). Originally created in English by Elias and colleagues [17], self-reported measures of foot preference are the basis of the Waterloo Footedness Questionnaire-Revised (WFQ-R). One of the most popular methods for figuring out foot preference is the WFQ-R. It has been utilized with a range of populations in the past, including healthy adults without disabilities [18], older adults [19], and people with neurological impairments [20].

The WFQ-R, however, has not been cross-culturally modified and translated for the Arabic population. Given the significance of doing so in clinical and behavioral contexts [4, 21], footedness self-reporting instruments need to be translated into several languages to accommodate a wide range of cultural contexts. Therefore, the goal of this study was to adapt the WFQ-R for usage in an Arabic-speaking community via translation and validation, as well as to analyze its psychometric qualities. The research team expected that the Arabic version of the Waterloo Footedness Questionnaire, WFQ-R-Ar, would be a viable measure of footedness among Arabic speakers, with high levels of internal consistency and test-retest reliability.

## Materials and methods

Translation and cross-cultural adaptation

The original WHQ questionnaire's developer, Dr. Lorin Elias, permitted the Arabic translation. The WFQ-R-Ar was translated and cross-culturally adapted in the following five steps according to recommendations made by Beaton and colleagues [22].

Forward translation

In step one, two bilingual, native Arabic speakers with a strong command of English, carried out the forward translation from English into Arabic separately. A written report for each translated version was presented to an expert committee together with the translators' observations and the justifications for decisions made regarding difficult problems. After settling disagreements between translators, the two forward-translated texts were combined into one Arabic version in step two. The expert committee also received a written report for the integrated version.

Back-translation and expert committee

In step three, two native English speakers independently completed the back-translation of the synthesized Arabic version into English and each submitted a report to the expert committee. In step four, a group of specialists in research methodology, language translation, and rehabilitation was assembled to assess all the translated versions and examine all the written reports provided. A prefinal version of WFQ-R-Ar was created after the committee resolved all differences following evaluation and review.

Pilot testing of the pre-final version

A total of 30 participants, all 18 years old and able to read, speak, and understand Arabic, took the pre-final WFQ-R-Ar version test in step five. Independent completion of the pre-final WFQ-R-Ar version was required from the participants. During the in-person interview, patients were invited to speak openly and honestly about the items' clarity, relevance, and any challenges they had in answering the questionnaire. The pre-final draft was modified slightly after the committee had read the notes and recommendations from the patients.

Participants and data collection

Participant recruitment was done in the community via advertisement posters and word of mouth, using a convenient sampling approach. Men and women aged 18 and up were eligible to participate from all throughout Saudi Arabia, and those who could read, write, and interpret Arabic were given priority. The study cohort included in the analysis consisted of the 290 remaining individuals after 30 of the 320 recruited participants withdrew from the study. The study's investigator contacted potential participants to participate. The study's objectives and methods were explained to patients who accepted the invitation, and they were also requested to read and sign a written consent form.

All study participants (n = 290) needed a demographic form to determine which foot was dominant. The footedness was evaluated using the following question, with the choices "right" and "left". It's like asking, "With which foot do you prefer to kick this ball in front of you?". The WFQ-R-Ar questionnaire was then administered to individuals twice at baseline and once within one week following the first test-retest reliability assessment. The patient's demographic data, which included age, gender, education, occupation, and dominant foot, were provided.

Questionnaire

The WFQ-R-Ar measures how often each foot is used via 13 questions answered on a 5-point Likert scale. A number between -2 and 2 is given to each response, with scores closer to 0 showing ambipedalism (the capacity to use both feet equally effectively), scores closer to -2 suggesting left-footedness, and scores of two indicating right-footedness. Respondents are classified as ambipedal, left-footed, or right-footed based on their total score, which ranges from -6 to +6, -7 or below, and +7 or more, correspondingly [18].

Data analysis

Participants' characteristics were analyzed using descriptive statistics. The foot dominancy was measured by the footedness question and WFQ-R-Ar. The participant's responses to the interview questions regarding the applicability and suitability of the scale to assess foot dominance were used to determine face validity. If the experts on the committee agreed that the scale was pertinent and suitable for assessing Arabic speakers' footing, then the content's legitimacy was established. More than or equal to 15% of respondents needed to provide the lowest or highest possible score for the scale to have a floor or ceiling effect [23].

Factor analysis using the direct oblimin rotation approach was used to determine whether or not the WFQ-R-Ar has construct validity by identifying and extracting components with eigenvalues larger than one, as defined by Kaiser [24]. The reliability of the WFQ-R-Ar was calculated using Cronbach's alpha, and a value of 0.7 was considered enough for determining reliability [23]. Using the interclass correlation coefficient for absolute agreement (ICC2,1), we determined that an ICC2,1 value of 0.70 or above was required to provide reliable test-retest results for the WFQ-R-Ar. The scale measurement error associated with the test-retest was analyzed using the standard error of measurement (SEM). An equation was used to determine the SEM: SEM = standard deviation × √(1 - ICC).

The construct was validated using hypothesis testing. Construct validity is the degree to which an instrument's score reflects what it is intended to reflect [23]. The expected correlations between the WFQ-R-Ar and the dominancy question that participants responded to were examined by Spearman's correlation coefficient (rs). It was determined that there were fair, weak, and little or no correlations based on the values of rs of >0.50, 0.50-0.35, and 0.35, respectively. According to the suggested quality requirements for measurement characteristics, fair correlation values >0.50 are regarded as acceptable correlations [23]. All data analyses were performed using IBM SPSS Statistics software for Windows, version 26 (IBM Corp., Armonk, NY, USA). The significance level was set at <0.05.

This article was previously posted to the medRxiv preprint server on April 27, 2020.

## Results

Two hundred and ninety Arabic-speaking participants took part in this investigation. Table 1 displays specific participant characteristics in detail.

**Table 1:** Basic characteristics of study participants SD: standard deviation

Characteristics	Mean (SD) or n (%)
Age (in years)	29.9 (11.3)
Sex	
Men	133 (46)
Women	157 (54)
Qualification	
Primary school or less	25 (9)
High school	103 (35)
Undergraduate	150 (52)
Graduate or more	12 (4)
Occupation	
Student	132 (45)
Working	101 (35)
Not working	57 (20)
Footedness	
Left-footedness	28 (10)
Right-footedness	262 (90)

The WFQ-R-Ar's translation and cultural adaptation were smooth and error-free operations. The question "Which foot would you use to help push a shovel into the ground?" (#9) was the only one that needed further explanation to be understood. Furthermore, by replacing acronyms (La/Ru) with genuine words (left always/right frequently), the subsequent cultural adaptation improved the respondent's understanding of the answer grid. In addition, people questioned after the pilot study said the questionnaire was clear, relevant, and appropriate for determining foot dominance. The testimony of these people attests to the WFQ-R-Ar's apparent validity. Also, the expert group has agreed on the usefulness and suitability of the WFQ-R-Ar for Arabic speakers to identify foot dominance. The analysis's satisfactory item completion rate and the absence of floor and ceiling effects further support the WFQ-R-Ar's content validity.

In terms of construct validity, The Kaiser-Meyer-Olkin test of sample size (p = 0.93), the sphericity test (p 0.001), and the determinant score (r = 0.112) all indicate that the distribution is not multicollinear. These three outcomes indicated that factor analysis was a viable option for the data. There's only one thing that accounted for 60.05% of the variance and had an eigenvalue greater than Kaisar's threshold of one (6.01), which was identified through factor analysis (Table 2).

**Table 2:** Factor structure of the WFQ-R-Ar WFQ-R-Ar: the Arabic version of the Waterloo Footedness Questionnaire-Revised

Factor	Total	Initial eigenvalue % of variance	Cumulative %	Total	After extraction % of variance	Cumulative %
1	6.01	60.79	60.05	5.58	55.79	55.79
2	0.82	8.18	68.23			
3	0.63	6.27	74.49			
4	0.50	5.04	79.53			
5	0.47	4.65	84.18			
6	0.41	4.13	88.31			
7	0.38	3.75	92.06			
8	0.29	2.92	94.98			
9	0.28	2.78	97.75			
10	0.26	2.25	100.00			

The factor extracted explained 55.79% of the variation after extraction. As a result, the WFQ-R-Ar has a single major dimension for all objects. Furthermore, each of the 10 items had a loading of greater than 0.5 on that single component, ranging from 0.56 to 0.82 (Table 3).

**Table 3:** Factor loading on each item of the WFQ-R-Ar α: alpha; WFQ-R-Ar: Arabic version of the Waterloo Footedness Questionnaire-Revised

Questionnaire (single factor)	Cronbach’s α	Items	Cronbach’s α if item deleted	Rotated factor loadings
Footedness	0.932	Item 1	0.924	0.82
		Item 2	0.926	0.82
		Item 3	0.924	0.78
		Item 4	0.925	0.77
		Item 5	0.923	0.77
		Item 6	0.922	0.74
		Item 7	0.923	0.73
		Item 8	0.926	0.73
		Item 9	0.925	0.71
		Item 10	0.927	0.56

The WFQ-R-Ar has excellent internal consistency with a Cronbach's alpha (α) of 0.93. Excellent test-retest reliability was found for WFQ-R-Ar stability, with an intra-class correlation coefficient (ICC) value of 0.94 (95% confidence interval (CI) = 0.93-0.95) (Table 4).

**Table 4:** Internal consistency, test-retest reliability, and the standard error of measurement α: alpha; WFQ-R-Ar: Arabic version of the Waterloo Footedness Questionnaire-Revised; ICC: intra-class correlation coefficient; CI: confidence interval; SEM, standard error of measurement

Scale	Cronbach’s α	Test	Retest	ICC	95% CI	SEM
WFQ-R-Ar	0.93	10.77 (9.4)	10.52 (9.6)	0.94	0.93-0.95	4.5

Also, the test-retest reliability scale SEM is 4.5 points. Furthermore, it was determined that the predicted association between the WFQ-R-Ar and the participant's dominancy response was significant (r = 0.48, p <0.001).

## Discussion

This study details the psychometric features of the WHQ-R-Ar, an Arabic version of the original WHQ that has been modified to fit local customs. Results show that the WFQ-R-Ar translation and cultural adaptation were effective. The WHQ-R-Ar's validity, internal consistency, test-retest reliability, and measurement error were all sufficiently high to recommend it for use in predicting footedness among Arabic-speaking adults.

The results of cross-cultural adaptation support WFQ-R-Ar's face validity. The WFQ-R-Ar was deemed relevant and acceptable for measuring footedness by a committee of experts who worked on the translation. Face validity was further reinforced by participant testimonials, which unanimously agreed that the WFQ-R-Ar was understandable, applicable, and well-suited to the purpose of measuring footedness. According to the assessments, the WFQ-R-Ar has sufficient content validity since it shows no floor or ceiling impact [23]. The WFQ-R-Ar was also put to the test of contemporaneous validity by correlating it to the question, "With which foot would you choose to kick this ball in front of you?". Clinical assessments of foot preference are comparable to the WFQ-R-Ar, indicating that it may be used to predict footedness.

Factor analysis was used to look into the WFQ-R-Ar's construct validity, and it was hypothesized that the WFQ-R-Ar would only consist of a single factor. The results of the factor analysis demonstrated that the WFQ-R items' Ar's are derived from a single common component. Every item extensively loaded this factor in the WFQ-R-Ar. The WFQ-R-Ar was responsible for a significant proportion (55.79%) of the variance. The WFQ-R-Ar's construct validity is confirmed by the factor analysis's overall findings, which also support the idea that this measure evaluates one underlying construct: footedness. The construct validity results from the present investigation are consistent with those previously reported [25].

Excellent internal consistency for the WFQ-R-Ar indicates that the scale's items are correlated, homogenous, and not redundant [26]. By removing each piece one at a time, the WFQ-R-Ar's Cronbach's alpha remained unchanged. According to Cronbach's alpha, all data are homogeneous and have strong correlations. The internal consistency and homogeneity of the scale would not be enhanced by removing any component.

The new research has better dependability than the Turkish study [27], Brazilian, Portuguese [20], and Chinese versions [28]. The SEM was 4.5 points (or 10%) when expressed as a percentage of the overall WFQ-R-Ar score. The SEM value and percentage in proportion to the total score demonstrate that the measurement error of the WFQ-R-Ar presented here is clinically suitable. Finally, almost 95% of the results fell within the agreement lines on the Bland-Altman plot, indicating adequate agreement between test and retest scores. As a consequence, trust in the WFQ-R-Ar was significantly bolstered.

Clinicians and researchers in Arabic-speaking countries now have a validated instrument to evaluate footedness, thanks to the efforts of this study to cross-culturally adapt and examine the measuring features of the WFQ-R-Ar. As was said, assessing footedness is a common first step for therapists when designing treatment plans. The evaluation of footedness by clinicians too often relies on a single inquiry. However, multi-item surveys are preferable for assessing lateral preference to binary ones.

For this analysis, we employed a translation of the WFQ-R into contemporary standard Arabic; dialects were not included. This translation was done in this way to encourage the usage of the current standard Arabic language in all Arabic-speaking countries, as it is understood across these regions. All the same, the participants in this research were all Saudis. So, before using the WFQ-R-Ar in other Arabic-speaking countries, a formal assessment of its measuring properties in the local context should occur. The WFQ-R-Ar has also been piloted on typically developing adults. The measuring properties of the WFQ-R-Ar need further study in different populations.

Some study limitations are that the study's sample consisted of participants who were at least 18 years old and could speak Arabic. This criterion may unintentionally exclude certain members of the community, which might introduce bias into the sampling process and reduce the results' applicability to a wider age range or individuals with less skill in Arabic. Participants' perceptions were used as data in the research. This raises the possibility of a biased reaction, as participants might provide answers that align with social desirability or misrepresent their true experiences. The study did not address the criterion validity of the WFQ-R-Ar by comparing its results to a gold standard measure or an established footedness assessment tool.

## Conclusions

The current study was conducted to explore the measuring characteristics of the WFQ-R-Ar's questionnaire by culturally adapting the original WHQ. The adaptation process went without a hitch, with minor changes to the original WHQ. The WFQ-R-Ar is a reliable and valid tool for measuring footedness in an Arabic-speaking population aged 18 years old. These WFQ-R-Ar measurement characteristics confirm the measure's relevance to all practical and academic purposes.
